# Status of insecticide susceptibility in *Anopheles gambiae *s.l. from malaria surveillance sites in The Gambia

**DOI:** 10.1186/1475-2875-8-187

**Published:** 2009-08-05

**Authors:** Martha Betson, Musa Jawara, Taiwo Samson Awolola

**Affiliations:** 1 Department of Infectious and Tropical Diseases, London School of Hygiene and Tropical Medicine, Keppel Street, London WC1E 7HT, UK; 2 Malaria Research Programme, Medical Research Council Laboratories, The Gambia, PO Box 273, Banjul, The Gambia; 3 Nigerian Institute of Medical Research, PMB 2013 Yaba, Lagos, Nigeria; 4 Department of Zoology, Natural History Museum, Cromwell Road, London SW7 5BD, UK

## Abstract

**Background:**

Vector control is an effective way of reducing malaria transmission. The main vector control methods include the use of insecticide-treated bed nets and indoor residual spraying (IRS). Both interventions rely on the continuing susceptibility of *Anopheles *to a limited number of insecticides. However, insecticide resistance, in particular pyrethroid-DDT cross-resistance, is a challenge facing malaria vector control in Africa because pyrethroids represent the only class of insecticides approved for treating bed nets and DDT is commonly used for IRS. Here baseline data are presented on the insecticide susceptibility levels of malaria vectors prior to The Gambian indoor residual spraying intervention programme.

**Methods:**

*Anopheles *larvae were collected from six malaria surveillance sites (Brikama, Essau, Farafenni, Mansakonko, Kuntaur and Basse) established by the National Malaria Control Programme and the UK Medical Research Council Laboratories in The Gambia. The mosquitoes were reared to adulthood and identified using morphological keys and a species-specific polymerase chain reaction assay. Two- to three-day old adult female mosquitoes were tested for susceptibility to permethrin, deltamethrin and DDT using standard WHO protocols, insecticide susceptibility test kits and treated papers.

**Results:**

All *Anopheles *mosquitoes tested belonged to the *Anopheles gambiae *complex. *Anopheles arabiensis *was predominant (54.1%), followed by *An. gambiae *s.s. (26.1%) and *Anopheles melas *(19.8%). *Anopheles gambiae *s.s. and *An. arabiensis *were found at all six sites. *Anopheles melas *was recorded only at Brikama. Mosquitoes from two of the six sites (Brikama and Basse) were fully susceptible to all three insecticides tested. However, DDT resistance was found in *An. gambiae *from Essau where the 24 hours post-exposure mortality was <80% but 88% for permethrin and 92% for deltamethrin.

**Conclusion:**

This current survey of insecticide resistance in *Anopheles *provides baseline information for monitoring resistance in The Gambia and highlights the need for routine resistance surveillance as an integral part of the proposed nation wide IRS intervention using DDT.

## Background

Malaria vector control, using either insecticide-treated nets (ITNs) or indoor residual spraying (IRS), relies on the continued susceptibility of *Anopheles *mosquitoes to a limited number of insecticides. Twelve insecticides from four classes (organochlorines, organophosphates, carbamates and pyrethroids) have been recommended for IRS [1,2], but only pyrethroids have been approved for treating bed nets. Since the mid-1950s, there have been numerous reports of reduced *Anopheles *susceptibility to DDT, malathion, fenithrotion, propoxur and bendiocarb, and resistance to all four classes of insecticides has been found in *Anopheles *species in different parts of Africa [3-12]. A much more recent development is that of pyrethroid resistance with cross-resistance to DDT, first reported in *Anopheles gambiae *from Côte d'Ivoire [13] and now widespread in West Africa. Pyrethroid-DDT cross-resistance presents a major challenge for malaria vector control in Africa because pyrethroids represent the only class of insecticides approved for treating bed nets and DDT is recommended for use in IRS [14].

Knockdown resistance (*kdr*) associated with a single point mutation in the gene encoding the voltage-gated sodium channel is a common mechanism of resistance to both pyrethroids and DDT. This mutation results in a leucine to phenylalanine substitution found predominantly in West Africa (*kdr*-w) or a leucine to serine substitution (*kdr*-e), which was originally identified in Kenya but is now found in several other African countries including Cameroon, Equatorial Guinea, Gabon, Angola and Uganda [15-18]. Both forms of *kdr *presumably function through reducing the affinity of DDT and pyrethroids for their target site on the sodium channel [15].

A recent retrospective analysis of malaria indices in the Gambia has shown a decline in both mortality and morbidity associated with malaria [19]. Although various factors could be responsible for the observed decline, the high coverage and usage of ITNs is thought to be a major contributing factor. To complement this effort, The Gambian National Malaria Control Programme (NMCP) is embarking on a nationwide IRS control intervention using DDT. This exercise requires information on the current susceptibility status of the major malaria vectors to insecticides recommended for malaria control, and to DDT in particular. This study presents baseline data in support of the NMCP prior to The Gambian IRS intervention.

## Methods

### Study sites

**Figure 1:**
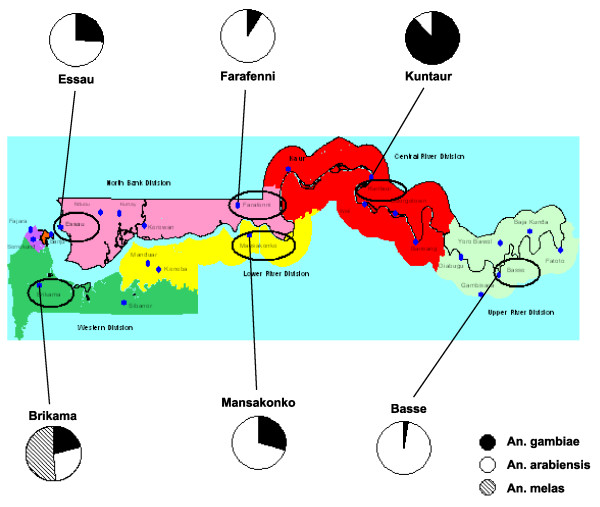
**Map of The Gambia showing the location of the larval collection sites**. Pie charts indicate relative proportions of *An. gambiae *s.s., *An. arabiensis *and *An. melas *found at each site. On the map green represents the Western Division, pink the North Bank Division, yellow the Lower River Division, red the Central River Division and light green the Upper River Division.

The study was carried out at six sites currently used for malaria surveillance in The Gambia: Brikama in the Western Division, Essau and Farafenni in the North Bank Division, Mansakonko in the Lower River Division, Kuntaur in the Central River Division and Basse in the Upper River Division (Figure 1).

### Mosquito collections

*Anopheles *larvae were collected from natural breeding sites such as ponds and puddles in July 2008. To avoid the collection of siblings, larvae were sampled from more than one (usually at least four) breeding sites. In addition, for the Farafenni collections larvae were collected on four separate occasions over a period of two-three weeks. The mosquitoes were reared to adulthood in the insectary at the Medical Research Council field site in Farafenni and identified using morphological keys [20,21] and a species-specific polymerase chain reaction assay [22].

### Insecticide susceptibility tests

Insecticide susceptibility tests were carried out using the standard WHO protocol [23], insecticide susceptibility test kits and impregnated papers. Two- to three-day old non blood-fed adult female *Anopheles *were tested. Batches of 20–25 mosquitoes were exposed to test papers impregnated with DDT (4%), permethrin (0.75%) and deltamethrin (0.05%). Controls included batches of mosquitoes from each site exposed to untreated papers. The knockdown effect of each insecticide was recorded every 10 minutes over the one-hour exposure period. Mosquitoes were then transferred to a recovery tube and provided with 10% glucose solution. Final mortality was recorded 24 hours post-exposure. All batches of insecticide-impregnated paper used were pre-tested on a laboratory strain of *An. gambiae *s.s. maintained at the insectary, which is known to be highly susceptible to pyrethroids and DDT. All susceptibility tests were carried out at 26–29°C and 74–82% relative humidity.

### Species identification

After performing the bioassays, all mosquitoes were given a unique identification number and then were stored individually over desiccated silica gel for later identification using morphological keys [20,21]. Samples belonging to the *An. gambiae *complex where further identified using a species specific polymerase chain reaction (PCR) assay [22]. Briefly, genomic DNA was extracted from each mosquito using the Gentra Puregene DNA purification kit (Qiagen) according to the manufacturer's instructions. 1/100th of the genomic DNA from one mosquito was combined in a 25 ***μ***l reaction with 1× Taq reaction buffer (10 mM Tris-HCl, 50 mM KCl), 1 mM MgCl_2_, 6.25 ng primer GA, 12.5 ng primers UN and ME, 18.75 ng primer AR, 200 ***μ***M of each dNTP and 0.625 U Taq (New England Biolabs). 30 cycles of PCR were carried out, each cycle consisting of denaturation at 94°C for 30 s, annealing at 50°C for 30 s and extension at 72°C for 30 s.

### Data analysis

Data were analysed using Excel and the R^® ^statistical package, version 2.8.0. Results from the insecticide susceptibility bioassays were evaluated according to the recommendations of the WHO [23]. Fifty and 95% knock-down times (KDT_50 _and KDT_95_) were estimated by means of a log-time probit model using the Ldp Line^R ^software [24-26].

## Results

### Species composition

A total of 1250 *Anopheles *were assayed. Almost all (99.9%) belonged to the *An. gambiae *complex. The PCR analysis showed that *Anopheles arabiensis *was predominant (54.1%), followed by *An. gambiae *s.s. (26.1%) and *Anopheles melas *(19.8%). *Anopheles gambiae *s.s. and *An. arabiensis *were found at all six sites. *Anopheles melas *was recorded only at Brikama where it constituted about 50% of the mosquitoes collected. The relative proportions of *An. gambiae *s.s. and *An. arabiensis *varied between the different study sites (Figure 1). Mosquitoes from Farafenni and Basse were predominantly *An. arabiensis *(Farafenni: 91.1%; Basse: 97.1%). In contrast, most (88.3%) *Anopheles *from Kuntaur were *An. gambiae *s.s.. A chi-squared test indicated significant difference in relative proportions of *An. gambiae *s.s., *An. arabiensis *and *An. melas *between the different study sites (p < 0.0001).

### Insecticide assay

According to WHO recommendations, 98–100% mosquito mortality indicates susceptibility, 80–97% suggests potential resistance that needs to be confirmed, and < 80% mortality suggests resistance [23]. Based on these criteria, *An. gambiae *s.l. mosquitoes from Brikama and Basse showed complete susceptibility to all three insecticides, mosquitoes from Kuntaur were highly susceptible to DDT as were those from Mansakonko to deltamethrin (Table 1[27]). Although the number of mosquitoes assayed at Mansakonko and Kuntaur were low compared to other sites, the point estimates for percentage mortalities suggest potential resistance of *An. gambiae *s.l. from Kuntaur to permethrin and deltamethrin and of *An. gambiae *s.l. from Mansakonko to DDT and permethrin (Table 1). *Anopheles gambiae *s.l. from Essau were resistant to DDT (69.6% mortality; 95% CI: 59.5–79.7%), and were potentially resistant to permethrin and deltamethrin while *An. gambiae *s.l. from Farafenni showed potential resistance to all three insecticides (Table 1). Species analysis of the resistant mosquitoes indicated that all DDT-resistant mosquitoes were *An. arabiensis*, whereas pyrethroid resistant mosquitoes were both *An. arabiensis *and *An. gambiae *s.s.. In Essau there was a significant difference in the proportion of *An. arabiensis *mosquitoes in DDT-resistant versus non-DDT-resistant *Anopheles *(two-tailed Fisher's exact test, p = 0.01). In all other cases, the number of surviving mosquitoes in each area was not significant so a logical conclusion to could not be drawn.

**Table 1:** Number of *Anopheles gambiae *s.l. mosquitoes tested and percentage mortality observed in insecticide susceptibility tests.

Study site	Variable	DDT (4%)	Insecticide Permethrin (0.75%)	Deltamethrin (0.05%)
**Brikama**	No. exposed	160	150	155
	No. dead (%)	160 (100)	150 (100)	155 (100)
	95% CI^$^	97.8, 100	97.6, 100	97.6, 100
	% alive An. arabiensis*	NA^†^	NA	NA
				
**Essau**	No. exposed	80	63	66
	No. dead (%)	57 (71.3)	56 (88.9)	61 (92.4)
	95% CI	60.0, 80.8	78.4, 95.4	83.2, 97.5
	% alive *An. arabiensis*	100	85.7	75.0
				
**Farafenni**	No. exposed	78	76	81
	No. dead (%)	71 (91.0)	72 (94.7)	75 (92.6)
	95% CI	82.4, 96.3	87.1, 98.5	84.6, 97.2
	% alive *An. arabiensis*	100	75.0	83.3
				
**Mansakonko**	No. exposed	22	18	22
	No. dead (%)	21 (95.5)	16 (88.9)	22 (100)
	95% CI	77.2, 99.9	65.3, 98.6	84.6, 100
	% alive *An. arabiensis*	100	50	NA
				
**Kuntaur**	No. exposed	38	41	38
	No. dead (%)	38 (100)	40 (97.6)	37 (97.4)
	95% CI	84.6, 100	92.5, 100	91.9, 100
	% alive *An. arabiensis*	NA	100	NA
				
**Basse**	No. exposed	22	19	20
	No. dead (%)	22 (100)	19 (100)	20 (100)
	95% CI	84.6, 100	82.4, 100	83.2, 100
	% alive *An. arabiensis*	NA	NA	NA

^$ ^95% confidence interval calculated by the exact method

* % of surviving mosquitoes which were *An. arabiensis*. Other surviving mosquitoes were *An. gambiae *s.s.

^† ^NA = not applicable/available

### Knockdown effect

**Figure 2:**
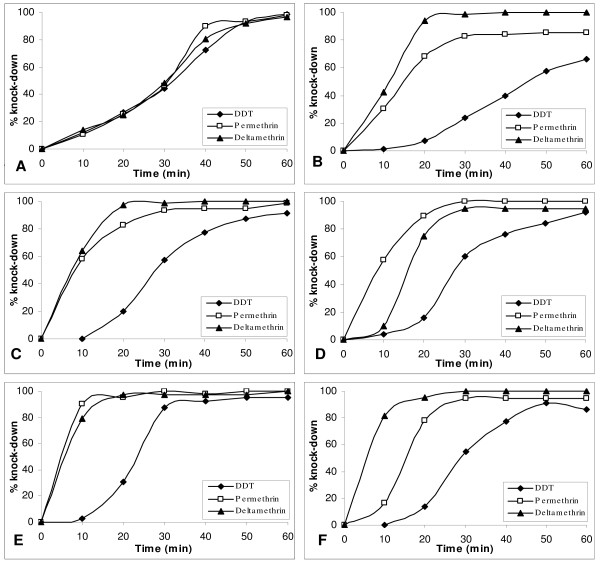
**Knockdown rate of unfed female *Anopheles gambiae *s.l. mosquitoes**. from Brikama (A), Essau (B), Farafenni (C), Mansakonko (D), Kuntaur (E) and Basse (F) exposed to pyrethroid and DDT treated papers.

The knockdown effect of the three insecticides determined over a one-hour period indicated that, in all cases apart from Brikama, knockdown was more rapid for pyrethroid insecticides than DDT (Figure 2). All mosquitoes showed over 90% knock-down within the one hour exposure period, apart from Essau mosquitoes exposed to DDT and permethrin and Mansakonko mosquitoes exposed to DDT (Figure 2). Exposure times which resulted in 50% and 95% knockdown (KDT_50 _and KDT_95_) estimated for each insecticide using a log-time probit model were consistent with the DDT resistance observed in *An. gambiae *s.l. from Essau: the KDT_50 _and KDT_95 _for DDT were higher for mosquitoes from Essau than for mosquitoes from other sites (Table 2). *Anopheles gambiae *s.l. from Essau also showed a higher KDT_95 _for permethrin than *An. gambiae *s.l. from other sites. KDT_50 _and KDT_95 _for deltamethrin did not vary substantially between mosquitoes from the different study sites (Table 2).

**Table 2:** Knockdown times (KDTs) for *Anopheles gambiae *s.l. in The Gambia after exposure to different insecticides.

Insecticide	Study site	**n**^**@**^	KDT_**50 **_(CL^**#**^)	KDT_**95 **_(CL^**#**^)
**DDT (4%)**	Brikama	166	26.1 (17.0–34.1)	68.3 (67.9–148.9)
	Essau	80	46.1 (42.4–50.8)	126.6 (102.1–174.3)
	Farafenni	80	28.7 (26.3–30.8)	63.9 (56.9–75.1)
	Mansakonko	22	30.1 (25.5–34.2)	64.3 (53.1–92.3)
	Kuntaur	39	22.7 (20.1–25.1)	47.0 (41.2–56.7)
	Basse	25	28.6 (24.6–32.5)	67.5 (55.4–93.4)
				
**Permethrin (0.75%)**	Brikama	150	27.6 (21.6–31.8)	52.3 (47.8–74.7)
	Essau	63	14.3 (10.9–17.3)	85.0 (64.5–132.0)
	Farafenni	76	6.9^$^	54.7^$^
	Mansakonko	18	15.0 (10.9–18.6)	42.6 (33.6–63.2)
	Kuntaur	41	1.0*	17.2*
	Basse	19	9.2 (5.2–11.8)	21.9 (16.9–40.7)
				
**Deltamethrin (0.05%)**	Brikama	155	25.1 (17.1–32.1)	67.8 (64.0–131.4)
	Essau	66	10.8 (9.4–12.0)	21.7 (18.9–26.7)
	Farafenni	88	8.3 (6.6–9.6)	18.7 (16.2–23.6)
	Mansakonko	22	5.6*	16.8*
	Kuntaur	38	3.3 (0.2–6.8)	24.3 (16.8–50.6)
	Basse	20	16.3 (12.7–19.5)	41.3 (33.9–55.8)

^@ ^Total number of mosquitoes exposed to each insecticide.

^# ^95% confidence limits

^$ ^The linear log-time probit model is not a good fit to the data in this case. Therefore the estimates of KD_50 _and KD_95 _are unlikely to be accurate and the confidence limits could not be estimated

* Lower and upper confidence limits could not be estimated due to large g value [25]

## Discussion

The species composition of *Anopheles gambiae *complex found in this study did not differ from previous records in the Gambia [28-30]. The preponderance of *An. arabiensis *contrasts previous findings but reflects the fact that larvae were sampled at the beginning of the rainy season when conditions tend to be drier and unsuitable for *An. gambiae *s.s.. The absence of *An. melas *in samples from Essau, Farafenni and Mansakonko, where there is brackish water in the flood plains, may be attributable to the fact that larvae were mostly sampled from pools and puddles rather from floodwater, the preferred breeding sites of *An. melas *[31].

The KDT_50 _and KDT_95 _for DDT in mosquitoes from the Essau area are relatively higher than those reported for susceptible reference strains [32-36], suggesting that a knockdown resistance mechanism could be operating in this mosquito population. The knockdown times for *Anopheles *from other sites were comparable to those published for *Anopheles *strains classified as susceptible to DDT, permethrin and deltamethrin [32-36].

The results of this investigation provide evidence of DDT resistance in *Anopheles *from Essau. DDT resistance was not detected at three sites (Basse, Brikama and Kuntar) but data from two other sites (Farafeni and Mansakonko) suggests the need for continued surveillance. The presence of DDT resistance at Essau is not surprising given the numerous reports of insecticide resistance in other West African countries including neighbouring Senegal [8,37,38]. A study conducted in a group of villages on the south bank of The River Gambia near Mansakonko in the early 90s [30] showed evidence of increased glutathione-S-transferase in the mosquito population with a link to DDT resistance. A recent survey of insecticide resistance in mosquitoes collected from Jangjangbureh (Georgetown), 11 miles from Kuntaur, found no evidence of resistance in anopheline mosquitoes, though marked resistance was found in culicine species (TSA, unpublished results).

In The Gambia, low levels of DDT residues have been found in soil sampled from various sites across the country [39]. It is possible that the past use of DDT and current use of pyrethroids have selected for resistance in *Anopheles*. The DDT and permethrin resistance observed at Essau is not suprising as similar findings have been reported in villages in neighbouring Senegal [8,37,38]. The overall level of DDT resistance recorded in The Gambia is low when compared to other West African countries. However, the information also provides an early warning of the development of insecticide resistance.

In Africa, DDT and pyrethroids resistance has been reported in the S and M molecular forms of *An. gambiae *s.s. and in *An. arabiensis *but not in *An. melas *[33,35,40-45]. The underlying resistance mechanism in most cases has been the *kdr *mutation [16,18,46-49]. In addition, metabolic resistance mechanisms have been implicated in reduced susceptibility to insecticides in both species [15,46,50,51]. In the present study, resistance to DDT was only detected in *An. arabiensis*, whereas resistance to pyrethroids was detected in both *An. arabiensis *and *An. gambiae *s.s.. Athough resistance could only be established at one site, these results have a couple of potentially interesting implications. Firstly, if DDT resistance is only found in *An. arabiensis *levels of resistance in *Anopeheles *mosquitoes may vary depending on the season reflecting the relative abundance of *An. arabiensis *and *An. gambiae *s.s.. Secondly, *kdr *mutation may play a role in *An. arabiensis *resistance at Essau where resistance was established. However, the absence of pyrethroid-DDT cross resistance in *An. gambiae *in this area suggest the possible role of a metabolic-based resistance mechanism.

Ideally, before any insecticide-based control activities are introduced, the levels of insecticide resistance in the main malaria vector should be assessed to provide information for measuring the effectiveness of the intervention. However, in most sub-Saharan African countries, insecticide resistance monitoring is given a low priority by the National Malaria Control Programmes. With the take-off of the Gambian IRS programme, it is important that a resistance management strategy is put in place that includes routine monitoring of insecticide resistance and investigation of alternative insecticides for IRS. The present study sites provide ideal focal points for malaria surveillance as insecticide resistance data can be directly linked to both epidemiological and clinical data.

## Conclusion

This study is the first to report DDT resistance based on bioassay tests on the *An. gambiae *complex from The Gambia. The data provide baseline information on resistance levels in *Anopheles *before the Gambian health authority embarks on an IRS campaign using DDT. A routine resistance surveillance and management strategy should be introduced as an integral part of the proposed nationwide IRS intervention.

## Competing interests

The authors declare that they have no competing interests.

## Authors' contributions

MB undertook the field and laboratory work and drafted the manuscript. MJ coordinated the field activities and assisted with revision of the manuscript. TSA conceived of and designed the study, and revised the manuscript. All authors read and approved the final manuscript.
